# Prevalence Studies on CKDu Need Stringent Reporting on Outcomes to Enhance Comparability

**DOI:** 10.3390/ijerph17186877

**Published:** 2020-09-21

**Authors:** Kristina Jakobsson, Jason Glaser, Catharina Wesseling

**Affiliations:** 1 School of Public Health and Community Medicine, Institute of Medicine, University of Gothenburg, 405 30 Gothenburg, Sweden; 2 La Isla Network, Washington, DC 20009, USA; jason@laislanetwork.org (J.G.); inekewesseling@gmail.com (C.W.); 3 Unit of Occupational Medicine, Institute of Environmental Medicine, Karolinska Institute, 171 77 Stockholm, Sweden

**Keywords:** CKD, CKDu, DEGREE protocol

## Abstract

Prevalence studies on Chronic Kidney Disease of unknown etiology (CKDu) need stringent reporting on outcomes following existing guidelines. Only by doing so, the much-needed comparisons between occupations, regions and climates for the elucidation of the etiology/etiologies of CKDu, and subsequently for its prevention, are possible. We, here, comment on methodological issues in a recently published study on rice farmers from West Java, Indonesia.

Comment on Fitria et al.: Environmental and Occupational Risk Factors Associated with Chronic Kidney Disease of Unknown Etiology in West Javanese Rice Farmers, Indonesia [1].

Chronic Kidney Disease (CKD) not related to diabetes, hypertension or other specific etiology is increasingly taking a toll on manual laborers in hot climates. In Central America, the evidence for work-related heat stress as a driving factor has accumulated [2], even if other risk factors have not been ruled out. In South Asia there is hitherto limited evidence for any specific etiology, although substantial investigations with a focus on exposure to agrichemicals have been performed. In contrast, the heat stress hypothesis has not been properly explored in occupational settings in South and Southeast Asia.

In this first study on CKD and CKDu in Indonesian rice farmers, the authors enrolled male farmers from two villages with different geographical locations. The authors intended to use the DEGREE protocol for prevalence studies on CKDu [3].

Notably, this protocol, as well as a taskforce from the International Society of Nephrology ISN [4], recommends reporting findings separately for estimated glomerular filtration rate (eGFR) and for proteinuria/albuminuria. This is also in line with the new 2017 KDIGO classification scheme [5]. Fitria et al. instead present their data using a now obsolete classification that combines eGFR and proteinuria into five categories. For CKDu this classification is especially problematic since CKDu presents with little or no proteinuria until advanced stages [4], in stark contrast to diabetes and hypertensive nephropathy.

For investigations in a population that may be at risk of CKD and CKDu it is of importance to describe the distribution of renal function, not solely report dichotomies or broad categories. Using the combined category scale, the authors reported subjects with eGFR in the range 60–89 as well as >90 mL/min/1.73 m^2^ and proteinuria as CKD, and possibly also CKDu. In contrast, there was no information on subjects with eGFR in the range 60–89 without proteinuria. Thus, the prevalence of mildly decreased eGFR and possible CKDu will inevitably be distorted. Moreover, there is no possibility to compare the results from this study to prevalence studies following the DEGREE recommendations, and most studies in occupational groups from Central America.

A second methodological concern is the need for adequate timing for exposure assessment for chronic diseases such as CKD and CKDu, which are evolving over an extended time period. Unlike most previous studies, the authors laudably engaged in retrospective assessment of pesticide exposure (albeit at the end reporting the many different pesticides used by the farmers only as a single toxic entity of insecticides). In contrast, their WetBulb Globe Temperature (WBGT) measurements in the two locations were performed during the week when the subjects were interviewed. Contextual long-term information on heat and humidity, available from existing weather stations or other sources, is also needed. One week of measurements cannot characterize climatic conditions that might differentiate between the two villages. It is of importance to address external heat load over the farming seasons, in addition to the internal heat generation at work (in this study represented by degree of mechanization). Regardless, the WBGT results should have been reported. There may well be limited climatic differences between the two locations, but this study does not add information neither for, nor against, heat stress as a potential risk factor for kidney damage.

The method of farming, and its presumed associations with workload, is also potentially informative and requires further characterization, as well as an exploration of the risks associated with modern vs. traditional farming practices. In fact, the DEGREE board is working on a protocol to effectively assess workload demands so they are more comparable within and between studies in occupational settings.

In summary, further enhanced reporting from this study can likely give valuable information for understanding CKD and CKDu in Indonesian rice farmers, but we strongly urge the authors, as well as other researchers, to report data following existing guidelines. Only by doing so, the much-needed comparisons between occupations, regions and climates for elucidation of the etiology/etiologies of CKDu, and subsequently for its prevention, are possible.
